# SRF: a seriously responsible factor in cardiac development and disease

**DOI:** 10.1186/s12929-022-00820-3

**Published:** 2022-06-09

**Authors:** Anushka Deshpande, Prithviraj Manohar Vijaya Shetty, Norbert Frey, Ashraf Yusuf Rangrez

**Affiliations:** 1 Department of Internal Medicine III, Cardiology and Angiology, University Medical Center Schleswig-Holstein, Campus Kiel, Kiel, Germany; 2 Department of Cardiology, Angiology and Pneumology, University Hospital Heidelberg, Heidelberg, Germany; 3 DZHK (German Centre for Cardiovascular Research), Partner site Hamburg/Kiel/Lübeck, Kiel, Germany; 4 DZHK (German Centre for Cardiovascular Research), Partner Site Heidelberg/Mannheim, Heidelberg, Germany

**Keywords:** Serum response factor, Heart, SRF cofactors, SRF regulators, Embryonic development, Cardiogenesis

## Abstract

The molecular mechanisms that regulate embryogenesis and cardiac development are calibrated by multiple signal transduction pathways within or between different cell lineages via autocrine or paracrine mechanisms of action. The heart is the first functional organ to form during development, which highlights the importance of this organ in later stages of growth. Knowledge of the regulatory mechanisms underlying cardiac development and adult cardiac homeostasis paves the way for discovering therapeutic possibilities for cardiac disease treatment. Serum response factor (SRF) is a major transcription factor that controls both embryonic and adult cardiac development. SRF expression is needed through the duration of development, from the first mesodermal cell in a developing embryo to the last cell damaged by infarction in the myocardium. Precise regulation of SRF expression is critical for mesoderm formation and cardiac crescent formation in the embryo, and altered SRF levels lead to cardiomyopathies in the adult heart, suggesting the vital role played by SRF in cardiac development and disease. This review provides a detailed overview of SRF and its partners in their various functions and discusses the future scope and possible therapeutic potential of SRF in the cardiovascular system.

## Introduction

**Fig. 1:**
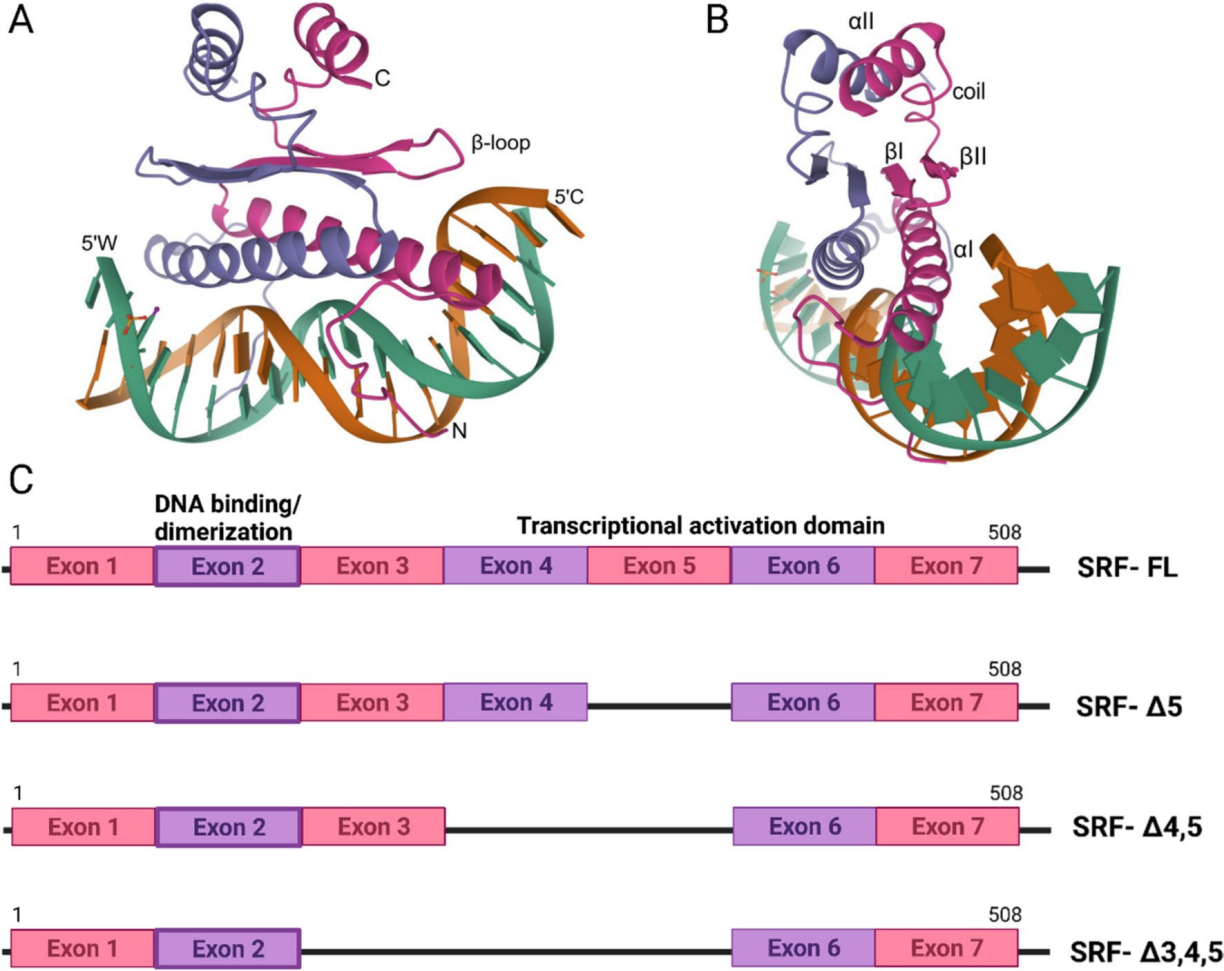
Genetic framework of SRF and its splice variants. **A** Crystal structure of the SRF core complexed with specific SRE DNA, **B** details of the α-helices and β-sheets; source—RCSB protein data bank (1SRS). **C** Graphical representation of the different splice variants with respect to the coding region of the SRF gene

The mechanisms involved in heart development in the embryo, heart maintenance in adulthood, the epithelial-to-mesenchymal transition (EMT) during gastrulation, and the transition of proliferating cardiac progenitor cells to terminally differentiated adult cardiomyocytes are tightly regulated through the synchronization of a myriad of signaling pathways by the interplay of various transcription factors [1, 2]. Serum response factor (SRF) is a transcription factor that plays an important role in multiple processes at different developmental stages of the pumping heart [3]. Located on chromosome 6p21.1, SRF spans 10,607 bp in humans and consists of seven exons. The 67-kDa SRF protein comprises 508 amino acids and consists of a transcriptional activation domain and an evolutionarily conserved MADS box with a DNA-binding domain, a dimerization domain and multiple cofactor-binding domains [4]. As the first member of the MCM1, Agamous, Deficiens and SRF (MADS) box family of transcription factors, SRF is highly conserved and ubiquitously expressed, serving as a regulatory protein of many intermediate regulatory complexes in myocyte and nonmyocyte lineages [5]. The N-terminal region of the MADS box is conserved and has an α-helix structure that is oriented in an antiparallel manner within homodimers to form a bipartite DNA-binding domain [6]. These α-helices align with the narrow major DNA groove and contact the phosphate backbone in the conserved CC(A/T)_6_GG sequence, which is called the CArG site, in the promoter [6]. This particular sequence is also called the serum response element (SRE) because the SRF protein recognizes its target gene promoters by the presence of this consensus motif [7]. The MADS box dimerizes upstream of the αI-helix through a structure composed of two β-sheets in each monomer that interacts with the same unit in its partner [6]. A second αII-helix is in the C-terminal portion of the MADS box, stacked upstream of the β-sheets, generating a stratified structure (Fig. 1A, B) [6].

The coding region of SRF consists of seven exons. Interestingly, SRF is expressed as four splice variants, with the largest variant containing all 7 exons [8]. SRFΔ5, which is alternatively spliced at the 5th exon, does not exhibit SRF activity due to the loss of 1/3 of its transactivation domain [8]. One variant is missing exons 4 and 5 (SRFΔ4, 5), and another variant has lost exons 3, 4 and 5 (SRFΔ3, 4, 5), as shown in Fig. 1C [8]. On the basis of the variants that do not exhibit SRF function, all splice variants that lack exon 5 are termed “dominant negative” isoforms because they do not show SRF activity [8–10]. However, the SRFΔ3 variant is not translated because of the premature termination codon, subjecting it to nonsense mRNA decay [11]. Although each SRF variant competes for binding to SRE sites, the full-length variant induces the maximum downstream gene activation [8].

SRF binds to SRE sites on the promoters of certain genes involved in cell contractility, movement and growth. The three groups of genes regulated by SRF are immediate early genes, muscle genes and growth-related genes [12]. Many of these genes respond to growth factor stimulation and tissue injury [13]. Interestingly, in addition to regulating cardiac- and muscle-specific genes, SRF undergoes self-regulation itself via the SRE in its own gene promoter [14]. Involved in various functions, such as actin treadmilling and mitochondrial dynamics, SRF tends to recruit other transcription factors at different times. Transcription factors known to interact with SRF include rat sarcoma-extracellular regulatory kinases (Ras-ERK), specific protein 1 (SP1), activating transcription factor 6 (ATF6), GATA4, NK2 homeobox 5 (Nkx2.5), and myogenic regulatory factors [13, 15, 16]. Thus, SRF is at the intersection of multiple signaling pathways controlling the expression of different target genes, thereby participating in cell growth and homeostasis in cardiomyocytes and in cell cycle regulation, cell growth, apoptosis and cell differentiation in other cell types, indicating the indispensable role it plays in the growth and development of an organism.

In this review, we focus on the roles played by SRF from development through adulthood by summarizing various studies depicting the importance of SRF at various stages of heart development. Digging deeper, we discuss various cofactors and modulators of SRF and explain how each of these candidates corresponds to cardiac homeostasis.

## Roles played by SRF in cardiac development

SRF is a master regulator of growth and development throughout the life of an organism. SRF is involved in cardiac trabeculation, chamber septation and myocardial wall thickness maintenance [17, 18]. The recruitment of smooth muscle cells (SMCs) to distal aorta is also facilitated by SRF [19]. With respect to cardiomyocytes, sarcomere and Z-disc organization is facilitated by this molecule [19]. In adults, SRF maintains cardiac equilibrium [3]. Notably, SRF might not affect all these processes directly; for example, many organogenesis processes are carried out by other molecules [20]. However, these organogenesis molecules are regulated by either SRF directly or by genes downstream of SRF [17, 21]. Overall, SRF plays an intrinsic role in embryonic stages, especially cardiac development and homeostasis. To understand the exact mechanisms by which SRF functions, this section has been divided into two subsections, one that presents the role played by SRF during embryonic development and one that describes SRF functions the postnatal stages [22].

### Prenatal stage

The stages of embryonic development maintain delicate balances between the expression of various molecules at precise locations during specific time points. Failed or slightly varied gene expression can trigger serious developmental defects, many leading to prenatal lethality. Specifically, deletion of SRF leads to detrimental effects in embryonic stages (Table 1). For example, SRF-null mice were smaller in size and presented with gastrulation defects, dying by embryonic day (E)8.5 [23]. Notably, these embryos were unaffected until E6.5, when they failed to form the primitive streak (ps) or mesoderm [23]. After E8.5, these embryos started disintegrating. Cre-Lox-based genetic recombination has provided unprecedented insights. Floxed *srf* expressed with Cre driven various cardiovascular gene promoters caused severely compromised sarcomere organization in embryos (Table 1) [18, 19, 24, 25]. Both structural and functional abnormalities in the primordial heart were characteristics of these embryos, which underwent in utero death [18, 19, 24, 25].

**Table 1:** Mouse models of SRF ablation with embryonic lethality

Embryonic day	Key heart characteristics	SRF KO Mutant	Consequences	Lethality	References
–	–	SRF null	• No change till E6.5	E8.5	[23, 42]
			• Small embryo		
			• Delayed development		
			• Inability to form Primitive Streak		
			• No mesodermal cells		
			• pr. of Pykontic TUNEL positive cells		
			• Impaired Gastrulation		
			• Fscn1↓, Crk1↓		
E 7.5–8.0	Fusion and formation of a single beating heart tube	Nkx 2.5 Cre	• Lack of Beating cells	–	[24]
			• Impaired sarcomerogenesis		
			• Hampered miRNA activity		
			• Cardiac α-actin ↓		
			• GATA 6 ↑, BMP4 ↑		
			• KCNMB1 ↓		
		βMHC Cre	• Sarcomerogenesis ↓	E10.5–13.5	[18]
			• Cell Survival ↓		
			• Apoptosis ↑		
			• Blood accumulation		
			• Enlarged ventricular lumen		
			• Impaired cell–cell interaction		
		αMHC Cre	• Chamber dilation	E11.5	[25]
			• Cardiac insufficiency		
			• Poorly developed interventricular groove		
			• Sarcomere organization ↓		
			• Apoptosis ↑		
			• Cardiac, skeletal, SMC α-actin ↓		
			• heartbeat stops around E11.5		
E 8.5–9.0	Heart tube undergoes looping followed by bulging of the heart regions	SM22α Cre	• Normal till E8.5	E11.5	[19]
			• Restricted growth		
			• Abnormal cardiac trabeculation		
			• Reduced vascular SMC recruitment to the dorsal aorta		
	Instrumentation of regular heart beat		• Disorganised cardiac sarcomere		
			• Loss of intermediate filament bundles in vascular SMC		
			• Compromised Z disc structure		

This table summarizes various SRF deletion mouse models at different embryonic stages. Constitutive as well as developmental stage-specific deletion of SRF using specific promoter Cre lines resulted in lethal defects suggesting the indispensable role SRF plays during embryonic development

*KCNMA1; Potassium Calcium-Activated Channel Subfamily M Alpha 1, pr; presence, Crk; c*hicken tumour virus no. 10 [CT10] *r*egulator of *k*inase*, FSCN1;* Fascin Actin-Bundling Protein 1

Cardiogenesis involves a multitude of factors that cause cardiac progenitor mesenchymal cells to lose pluripotency and ultimately become terminally differentiated cells in the heart. Embryonic growth in vertebrates proceeds in an anterior to posterior (A–P) direction. This elongation of the A–P axis depends on the addition of new cells from the posterior end of an embryo, which is generated by a transient ps. In mice, the ps is developed by E6.5, but the initial evidence of ps is seen at Hamburger Hamilton (HH) stages 1–2 in avian embryos [22, 26]. Interestingly, the initiation of SRF expression coincides with ps formation, which directs the EMT, which is required for the invagination and migration of epiblast cells that form the mesoderm and endoderm [22, 23]. Newly formed endoderm cells displace hypoblast cells at the base of the embryo [27]. Notably, research has suggested that the meso- and endoderm are derived from a common precursor called the mesendoderm, wherein cells receive specification signals and differentiate into a meso- or endoderm cell type [28, 29]. In Xenopus embryos, inhibiting SRF activity led to the expression of mesendodermal genes in the ectoderm; however, SRF-null mouse embryos failed to develop mesodermal cells [23, 30]. These outcomes my indicate that SRF maintains balanced germ layer specification in a gradient-dependent fashion [30]. Identification of the factors underlying the SRF gradient is an interesting future direction. SRF expression in the ps and lateral plate mesoderm (primary heart field) in chick embryos at HH6 and mice on E7.5 hints at the significance of SRF expression in cardiogenesis [31, 32]. In addition, SRF is expressed in the neural groove, somites and the cardiac crescent. Cells from the SRF-expressing pericardial splanchnic mesoderm (derived from the lateral plate mesoderm) migrate to form endocardial heart tubes, which later fuse to form a single tube [31, 33]. High levels of SRF accumulates in the myocardium in this stage and later [31, 32]. The beating process, which starts after the fusion and initiation of looping, is defective in SRF-ablated mice. The looping of the heart tube and the formation of the secondary heart field, which involves cells that accumulate to form the outflow tract, are associated with SRF expression [17, 18].

The coronary vasculature is primarily differentiated from proepicardial progenitor cells, which also necessarily express SRF to differentiate into SMCs [34, 35]. T-box transcription factor 18 (Tbx18) regulates the differentiation of SMCs from epicardial progenitor cells in the coronary vasculature via SRF/myocardin-dependent repression activity [36]. Similarly, transcription factor 21 (TCF21) inhibits SRF-myocardin DNA binding [37]. Rho kinase activity is also SRF-dependent. Host chick embryos with a chimeric epicardium derived from quail proepicardium that was pretreated with the p160 Rho kinase inhibitor Y27632 failed to develop coronary vasculature [38]. With addition to their functions in vasculature and other tissues, proepicardial cells give rise to the endothelium, the fibroblast population in the heart, atrioventricular valve cells, the epicardium, and mesenchymal populations in the subepicardial layer [33]. The involvement of SRF in various stages of cardiac development is intriguing; however, further investigation is needed to understand how SRF regulates the activity of different cardiac progenitors.

### Postnatal stage

After birth, SRF continues to transcriptionally control muscle-specific gene expression to maintain cardiac homeostasis [3, 39–41]. Thus, deletion of SRF postbirth drives detrimental cardiac effects, as shown in Table 2. In studies in which SRF expression was ablated to various degrees and in a stage-specific manner, mutant mice presented with disrupted cardiomyocyte architecture, mitochondrial dysfunction, and abnormal heart size and were ultimately destined for cardiomyopathy that led to heart failure (Table 2) [3, 39, 40]. Dimerization of incompetent SRF mutants in the heart caused various structural defects that were evident postpartum and led to death due to cardiomyopathy and heart failure [41].

**Table 2:** Mouse models of SRF modulation at postnatal developmental stage

Gene manipulation	Activity starting point	SRF protein characteristics	Consequences	Lethality	References
α-MHC-SRF	E 7.5–8.0	Overexpression of SRF	• Cardiomyopathy	6 to 40 weeks post-birth	[40]
			• Cardiomyocyte hypertrophy		
			• Dilation of 4 chambers		
α-MHC-dmSRF	E 7.5–8.0	Hampered dimerization and DNA binding capacity	• Atrial and ventricular chamber dilation	9–12 days post-birth	[41]
			• Reduced ventricular wall thickness		
			• Smaller cardiomyocytes		
			• Reduced myofibrils		
			• Dilated cardiomyopathy		
α-MHC-Cre SRF KO	Tamoxifen inducible	Knock out of SRF	• Reduced left ventricular contractibility followed by enlargement	10 weeks after tamoxifen treatment	[39]
			• Gradual increase in heart size		
			• Disruption in cardiomyocyte cytoarchitecture		
			• Dilated cardiomyopathy		
*SrfF/F*	AAV-Cre at P1	Knockout of floxed SRF upon AAV-Cre treatment	• Loss of T-tubule	–	[3]
			• Reduction in cardiomyocyte size		
			• Hampered sarcomeric assembly		
			• Decreased mitochondrial size		
	AAV-Cre at P60		• Minor T-tubule defects	–	
			• Reduction in cardiomyocyte size		
			• No sarcomeric disorganization		

This table summarizes various mouse models that were generated to study the effects of SRF modulation during post-natal stages

## Roles played by SRF in cardiac signal transduction

Cell signaling is a complex process in which the activation of genes depends on certain stimuli that are converted into a cascade of responses for inducing precise cellular actions. Although SRF has been shown to be an important contributor to cell growth, proliferation and differentiation, many different signaling cascades are involved in efficiently inducing and regulating needed cellular action [9, 43–46].

### Cofactors

**Fig. 2:**
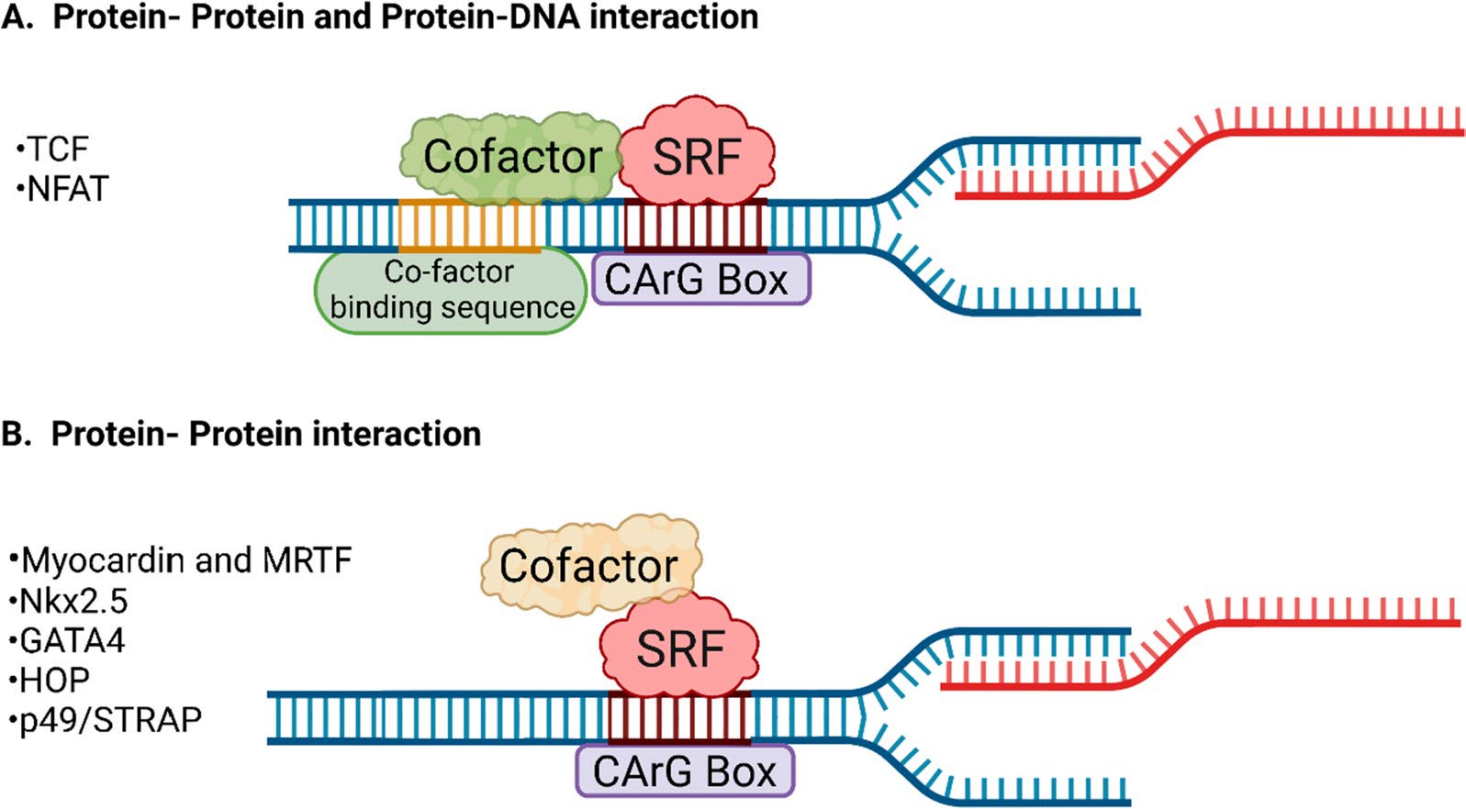
SRF-cofactor interaction. **A** Some cofactors can interact with the DNA backbone located adjacent to the CArG box binding site of SRF and associate with SRF directly. **B** Some cofactors interact directly with SRF because they cannot bind with DNA directly. *SRF* serum response factor; *TCF* ternary complex factor; *NFAT* nuclear factor of activated T cells; *MRTF* myocardin-related transcription factor; *Nkx2.5* NK2 homeobox 5; *HOP* homeobox protein; and *p49/STRAP* SRF-dependent transcription regulation-associated protein

Multiple cofactors have been shown to interact with SRF and induce its transcriptional activation. These cofactors interact with SRF either through protein–protein or protein–DNA interactions or both, as shown in Fig. 2. These cofactors have either a binding site adjacent to the SRE/CArG box, as shown in Fig. 2A, or bind directly to SRF without binding to the DNA backbone (Fig. 2B).

#### Ternary complex factor (TCF)

TCF proteins constitute a subfamily of proteins containing the E twenty-six (Ets) domain, including three members, Elk-1, Net and Sap-1 [47]. TCF family members show high sequence similarity in three regions: the Ets domain; the B box, which mediates interaction with SRF; and the C box, which includes the transactivation domain [47]. These SRF cofactors are involved in specific cellular activities.

*Proliferation* Upon stimulation by mitogens in eukaryotic cells, various biochemical changes are induced, pushing the cell toward proliferation by triggering signal transduction cascades involving mitogen-activated protein kinase (MAPK). MAPKs comprise three subfamilies, namely, the extracellularly responsive kinases (ERK), c-Jun N-terminal kinase (JNK) and p38 MAPK subfamilies [48]. The relevance of these proteins to this review is based on the SRE sites located upstream of many mitogen-inducible gene promoters that are important for MAPK signaling activation [49]. Interestingly, the Ets sequence is located adjacent to these SRE sites on the target gene promoters [47]. TCF-dependent pathways are involved in the activation of genes encoding proteins involved in the G0/G1 cell cycle phase transition or immediate early genes [50]. The C-fos promoter is the most extensively studied promoter with respect to SRF and TCF interactions [51]. Upon phosphorylation of the C box domain by MAPK, TCF is activated and translocated to the nucleus [47]. Through its Ets domain, TCF in the nucleus binds upstream of the C-fos promoter and, via its B box domain, binds to the SRF dimer at the SRE site [47].

*Stress response* In addition to growth or mitogen stimuli, stress triggers MAPK pathway activation [52]. Upon stress, p38 mitogen-activated protein kinases are triggered, leading to the transcription of appropriate genes involved in cellular responses. For example, in cardiomyocytes, atrial natriuretic factor (ANF) expression is triggered by SRF activation via the p38 MAPK activator MKK6-Glu [13]. Nevertheless, sequence mapping and interaction studies have revealed that p38 is a TCF-independent activator of SRF [13]. Interestingly, p38 phosphorylates ATF6, a likely potent binding partner of SRF [13]. The MAPK pathway undoubtedly plays a vital role in TCF-mediated SRF signaling, but MAPK family members may also act as independent activators of SRF. It is intriguing to consider how the members of each family activates SRF and the mechanisms through which these pathways intersect.

#### Myocardin and MRTF

Another crucial class of cofactors critical for SRF target gene activation is myocardin (MYOCD) and myocardin-related transcription factors (MRTFs). First discovered in the cardiovascular system, myocardin activity depends on SRF [53, 54]. Both myocardin and MRTF trigger CArG-dependent muscle transcription but only by interacting directly with SRF via a short peptide sequence that includes a glutamic acid region, as myocardin and MRTFs lack the sequence required for CArG binding [53, 54]. The SRF-binding region in myocardin and MRTFs resembles the B box, although it lacks direct Ets-binding region sequence homology, distinguishing this SRF-binding region from that of TCF-like cofactors [54, 55]. The dual regulatory mechanism of SRF on discrete extracellular stimuli is observable when Elk1 activation is triggered via MAPK signaling, and myocardin is dissociated from the MRTF-SRF complex and an Elk1-SRF complex with a transcription-inducing function is formed [56, 57]. Subsequent studies have shown that myocardin, a potent cofactor of SRF, is critical for various heart-related cellular functions.

*Cytoskeletal and contractile system* The N-terminus of myocardin and MRTFs contains three RPEL motifs that associate with actin, thereby enabling myocardin and MRTF responses to cytoskeletal signaling [56, 58]. SRF, in conjugation with myocardin and MRTF, induces the transcription of genes encoding proteins involved in actin microfilament dynamics and cellular mobility, including structural genes such as actin, effectors of actin turnover such as cofilin, and regulators of actin dynamics such as talin [50]. The transcriptional MRTF-SRF axis is both regulated by and is a regulator of monomeric G-actin level in a cell [59, 60]. When actin polymerization is slow, MRTFs are in an inactive state and bound to G-actin in the cytoplasm, indicating that MRTF is a G-actin-binding protein (G-ABP) [61]. Moreover, high levels of G-actin in the nucleus prevent MRTF-A from binding to SRF, resulting in another inhibitory feedback mechanism that tightly controls MRTF-A/SRF-mediated transcription [61]. An important regulator of this axis is α-actinin 2, and mutations in this gene increase the levels of monomeric actin, hindering MRTF nuclear localization[59, 62]. Additional elements involved in this axis are further explained in this review.

**Fig. 3:**
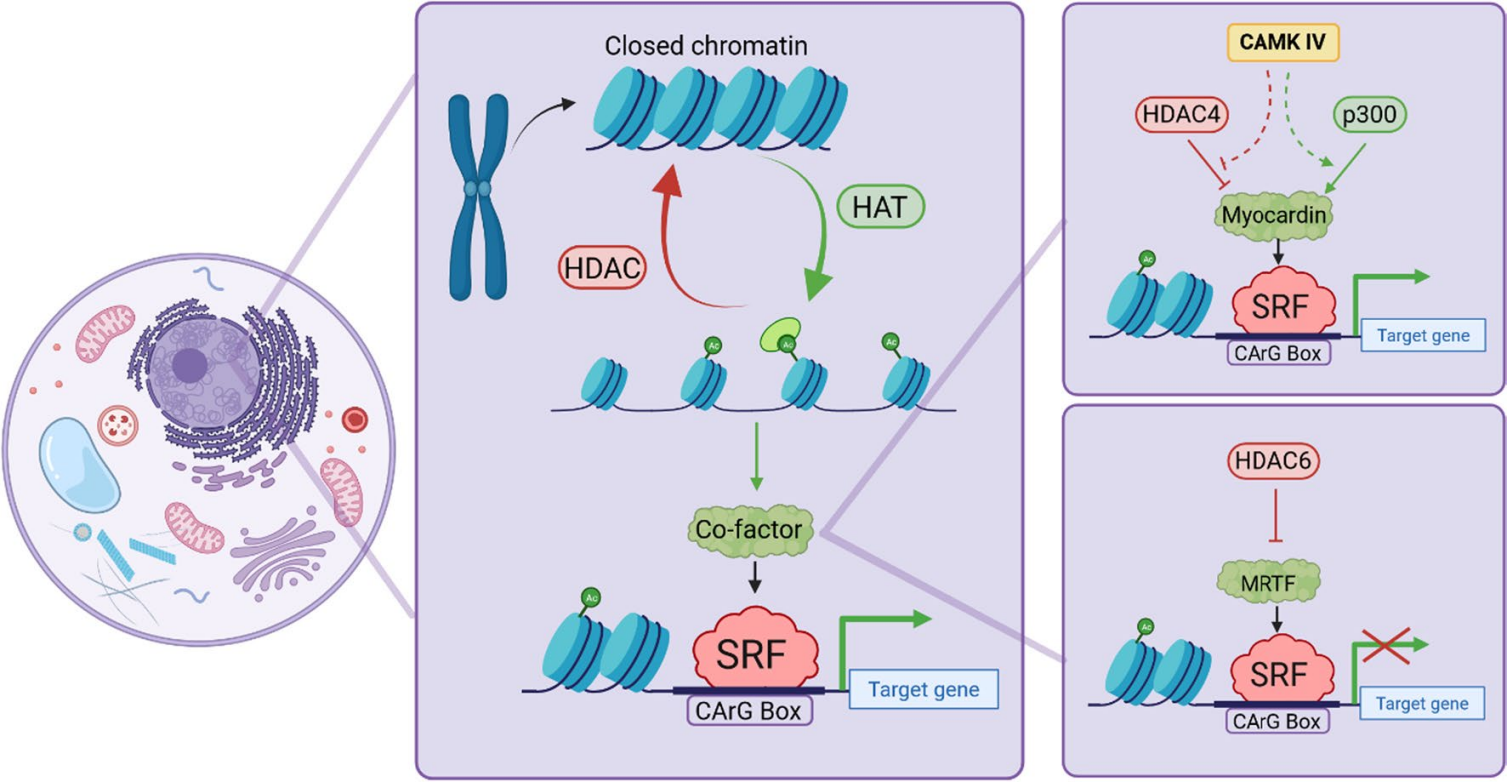
Chromatin remodeling and its implications on SRF signaling- Epigenetic changes play important roles in facilitating the activation of various transcription factors. After chromatin decondensation facilitated by histone acetyltransferases, the CArG site is available for SRF binding to induce transcription. This effect may be reversed by the action of histone deacetylases, resulting in chromatin condensation and rendering the CArG site unavailable for binding. Furthermore, mechanisms by which CAMK IV or HDAC6 regulates the SRF activation are observed; CAMK IV inhibits HDAC activation and promotes HAT activation, resulting in myocardin-SRF transcriptional activation. In contrast, HDAC6 prohibits MRTF-SRF activation, resulting in suppressed SRF transcriptional activity. *HAT* histone acetyltransferase; *HDAC* histone deacetylases; *SRF* serum response factor; and *MRTF* myocardin-related transcription factor

*Chromatin remodeling* Myocardin recruits chromatin-remodeling enzymes such as p300, a histone acetyltransferase (HAT), to enhance SRF-mediated target gene expression [63]. In contrast, interaction with class II histone deacetylases (HDACs) represses SRF-related target gene expression by repressing myocardin activity (Fig. 3) [63]. Moreover, myogenic repressor Kruppel-like factor 4 (KLF4) blocks SRF association with methylated histones and the CArG box by stimulating HDAC4 activity [64]. Calcium levels also play vital roles in HDAC4-SRF-mediated control of transcription [65]. Activation of calcium-calmodulin-dependent protein kinase IV (CaMK-IV) leads to dissociation of the HDAC4-SRF complex, after which HDAC4 is exported from the nucleus and is triggered by p300 to activate myocardin-SRF mediated transcription [65]. Thus, epigenetic control of SRF binding to chromatin plays a key role in -mediated regulation of SMC differentiation in response to pathophysiological stimuli [64]. Similarly, HDAC6 is a regulator of MRTF-A and SRF transcription in vascular SMCs [66]. Pathological vascular cells are marked by dedifferentiation. As HDACs critically regulate transcription, HDACs have been predicted to play a role in vascular pathogenesis [66]. Studies showed that both inhibition and ablation of HDAC6 activated SRF-activated genes, especially smooth muscle α-actin, which is necessary for vascular plasticity [66]. HDAC6 exerts its inhibitory effect by directly binding to MRTF-A and hindering MRTF-A-SRF axis in vascular SMCs (Fig. 3) [66].

*Embryogenesis* During embryogenesis, MRTF-A and B bind to SRF to activate the SRF downstream genes necessary for promoting required morphological changes [54, 55]. MRTF-A levels are significantly enriched in mesenchymal cells and muscle and epithelial cells during embryogenesis, while MRTF-B levels are higher in the branchial arch artery and neural structures, and therefore, these MRTFs affect morphogenesis at different sites [54]. Thus, myocardin and MRTF play crucial roles in maintaining the cardiovascular cytoskeleton by regulating epigenetic changes and actin treadmilling.

#### P49/STRAP

In 2004, p49/SRF-dependent transcription regulation-associated protein (STRAP), later named serum response factor-binding protein 1 (SRFBP1) by The Human Genome Organization (HUGO), was identified as a novel SRF-binding protein by Zhang et al*.* [67]*.* p49 activates ventricular myosin regulatory light chain 2 (MLC2v) and cardiac α-actin promoters in the presence of SRF but represses ANF promoter activity, which is regulated by the myocardin-SRF complex, with various effects on target activity [67]. Interestingly, the interactions of p49 are not limited to SRF alone and include SRF in complex with myocardin or Nkx2.5 [67].

*Cellular aging* Mitochondrial respiratory complex I is critical to the balance in NAD:NADH levels. Studies have suggested that the NADH dehydrogenase ubiquinone oxidoreductase subunit AB1 (NDUFAB1), a subunit of complex I, interacts with p49/STRAP [68]. Hindrance in the assembly of complex I may be a reason for the reduced cellular NAD levels upon ectopic overexpression of p49/STRAP [68]. Notably, p49/STRAP overexpression promoted histone H4 deacetylation, which was accompanied by downregulated peroxisome proliferator-activated receptor gamma coactivator 1-alpha (PGC 1α) and mitofusin 1 & 2 expression [69]. Mitofusins, which are regulated by PGC 1α, are important mitochondrial fusion proteins decisive in mitochondrial dynamics [70], and histone H4 acetylation is involved in chromatin remodeling function [71]. Hence, it has been postulated that p49/STRAP inhibits overall mitochondrial function, probably through SRF activity inhibition.

**Fig. 4:**
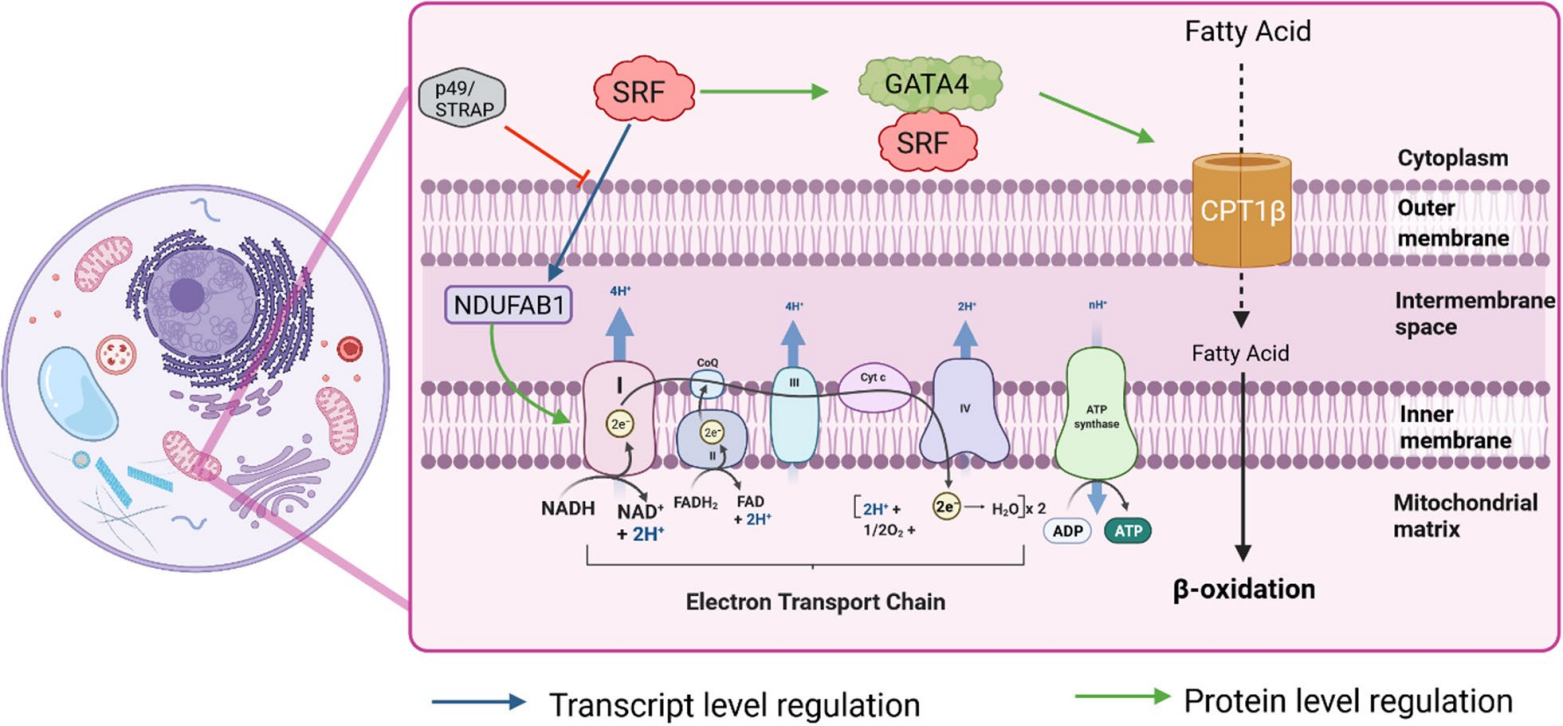
Effects of SRF and cofactors on mitochondrial gene expression—SRF transcriptional activation exerts an effect on mitochondrial gene expression. One of the major cellular energy-producing pathways is the electron transport chain, and SRF facilitates the activation of complex I via NDUFAB1 action, which is inhibited by p49/STRAP. SRF in association with GATA4 activates CPT1β, which is involved in fatty acid oxidation. *p49/STRAP* SRF dependant transcription regulation associated protein; *SRF* Serum response factor; *GATA4* GATA binding protein 4; *CPT1β* carnitine palmitoyltransferase 1β; *NDUFAB1* NADH dehydrogenase ubiquinone oxidoreductase subunit AB1

*Cytoarchitecture* Although primarily in the nucleus, p49/STRAP also resides in the cytoplasm close to actin molecules [72]. Overexpression of p49/STRAP led to a decline in both cell size and overall actin expression [72]. Transgenic mice with increased p49/STRAP expression presented with severe body malformations [72]. These mice exhibited reduced expression of SRF and other related muscle-specific genes (Fig. 4) [72].

In a comparison of young vs. old mice, p49/STRAP expression was higher in the latter [72]. This change combined with the aforementioned findings point to the role played by p49/STRAP in regulating cellular aging by negatively regulating SRF activity and thus modulating mitochondrial and cytoskeletal dynamics.

#### Nkx2.5 and GAT4

A cofactor of SRF, Nkx2.5, is also known as a cardiac-specific homeobox gene. Interaction of Nkx2.5 with SRF leads to activation of the cardiac α-actin promoter, and this activation peaks when GATA4 recruitment is highest [16, 21, 73, 74]. Nkx2.5 can bind directly to DNA, and even when the DNA-binding domain in the Nkx2.5 molecule is occupied, Nkx2.5 can still be recruited by SRF [16]. The SRF-Nkx2.5 complex can readily activate a promoter, but GATA4 causes a conformational change in the Nkx2.5 protein, displacing its inhibitory domain and dramatically increasing promoter activity [16].

*Mitochondrial dynamics* Several studies have highlighted the importance of mitochondrial gene expression in cardiomyocytes that is induced via the GATA4-SRF interaction. Transcriptional regulation of mitochondrial proteins depends on nuclear transcription factors [15]. In this context, the interaction of GATA4 and SRF is particularly important because it leads to the high expression of a heart mitochondrial protein, carnitine palmitoyltransferase 1β (CPT1β), which primes fatty acyl molecules for transportation into the mitochondria, as depicted in Fig. 4 [15]. This mechanism suggests that GATA4 and SRF mutually control the robust activation of mitochondrial proteins, in addition to the structural proteins, in cardiomyocytes; however, additional studies are needed to gain further mechanistic insights. Overall, Nkx2.5 alone or together with GATA4 constitutes an important regulatory mechanism that determines the extent of SRF-mediated gene expression.

#### Nuclear factor of activated T cells (NFAT)

Activation of α-actin in SMCs is induced by SRF in conjunction with another factor called NFAT. An overlap in the binding sites of SRF and NFAT on the α-actin promoter has been identified, and mutations of any of these sites reduced α-actin expression [75]. The NFAT-c3-SRF complex (C-terminal interaction) has been proposed to be one of the necessary factors for the activation of α-actin in SMCs [75]. Overall, NFAT in its phosphorylated form is located in the cytosol. In response to Ca^2+^, NFAT is dephosphorylated by calcium-dependent phosphatase and calcineurin and is translocated to the nucleus, where it interacts with SRF and enhances α-actin activation [75, 76].

As mentioned in the previous sections, the activation of α-actin in SMCs depends on a myocardin-SRF-induced signaling cascade. Therefore, we speculate that NFAT-SRF activity may parallel myocardin-SRF activation and that both pathways may be required for full gene activation. Interestingly, the Calcineurin-NFAT pathway is a major signaling mechanism in cardiac hypertrophy and heart failure [77]. Hence, an unidentified link between cardiac hypertrophy and heart failure caused by overexpression of SRF may be involved, and the likely dependence of this missing link on NFAT signaling needs to be explored further.

#### Homeobox protein (HOP)

HOP is a homeodomain protein expressed in the early development stages of the heart and is also expressed in postnatal stages [78, 79]. Although it is a homeodomain protein, it cannot bind to DNA because key amino acids are missing. HOPs physically interact with or bind to SRF and inhibit SRF-mediated cardiac-specific gene transcription [79]. HOP presumably blocks the myocardin-SRF-mediated transcriptional activation of the SM22α ANF promoter. Ironically, HOP is activated directly downstream of Nkx2.5. Whether the Nkx2.5-HOP axis is involved in a feedback mechanism regulating the level of SRF activation is an interesting but unknown possibility [78]. In addition, HOP has been suggested to recruit HDACs and form complexes with HDAC2 to inhibit SRF activity; this possible recruitment may be a reason for HOP-mediated cardiac hypertrophy [80]. Genetic ablation of HOP expression resulted in an increase in the expression of SRF-mediated genes and increased proliferation of cardiac myocytes in newborn mice [79]. Hence, HOP is considered a critical regulator of SRF that maintains the balance between differentiation and proliferation.

### Regulators

As explained in Table 3, multiple molecules exert regulatory effects on SRF signaling via their interactions with one or more cofactors.

**Table 3:** SRF regulators and cofactors

Regulators	Cofactors	Effect on SRF activity	Cell function	Affected stages	References
Rho	Myocardin & MRTF	Positive	Epicardial cell motility	Embryonic	[87]
			Cardiomyocyte lineage	Adult	[84]
			Angiogenesis	Adult	[88, 89]
			Actin dynamics	Adult	[50]
			Cell Cell contact	Adult	[84]
Myozap	Myocardin & MRTF	Positive	Stress response	Adult	[90, 92]
Dysbindin and Rnd1	Myocardin & MRTF	Positive	Hypertrophy	Adult	[90, 93, 95]
TGFβ	Myocardin & MRTF	Positive	Actin dynamics	Adult	[85, 98]
			Cardiomyocyte lineage	Adult	[104]
CAP2	Myocardin & MRTF	Negative	Actin dynamics	Adult	[106]
STARS	Myocardin & MRTF	Positive	Actin dynamics	Adult	[107, 108]
			Stress response	Adult	[109]
			Contractility	Adult	[110]
FHL	Myocardin & MRTF	Negative	Inhibition of angiogenesis	Embryonic	[111]
			Stress response	Adult	[112]
HAT	Myocardin & MRTF	Positive	Chromatin remodeling	Embryonic and adult	[63]
HDAC4	Myocardin & MRTF	Negative	Inhibition of Chromatin remodeling	Embryonic and adult	[63]
HDAC6	Myocardin & MRTF	Negative	Vascular SMC dedifferentiation	Embryonic	[66]
GATA	Nkx2.5	Positive	Mitochondrial dynamics	Adult	[15]
Calcinurin	NFAT	Positive	Smooth muscle actin	Adult	[77]
-	HOP	Negative	Cardiac morphology	Embryonic	[79]
HDAC2	HOP	Negative	Cardiac hypertrophy	Adult	[80]
NDUFAB1	p49/STRAP	Negative	Cellular ageing	Adult	[68]
YY1	–	Negative	Muscle specific gene inactivation	Embryonic and adult	[113]
Titin	–	Negative	Mechanical sensor	Adult	[114]
TRIM24	**–**	Positive	Cardiomyocyte hypertrophy	Adult	[115]
TRIM32	**–**	Negative	Inhibition of Cardiomyocyte hypertrophy	Adult	[115]

Summary of the effects of regulators and cofactors on SRF in particular and on cardiovascular system in general

#### Rho GTPases

**Fig. 5:**
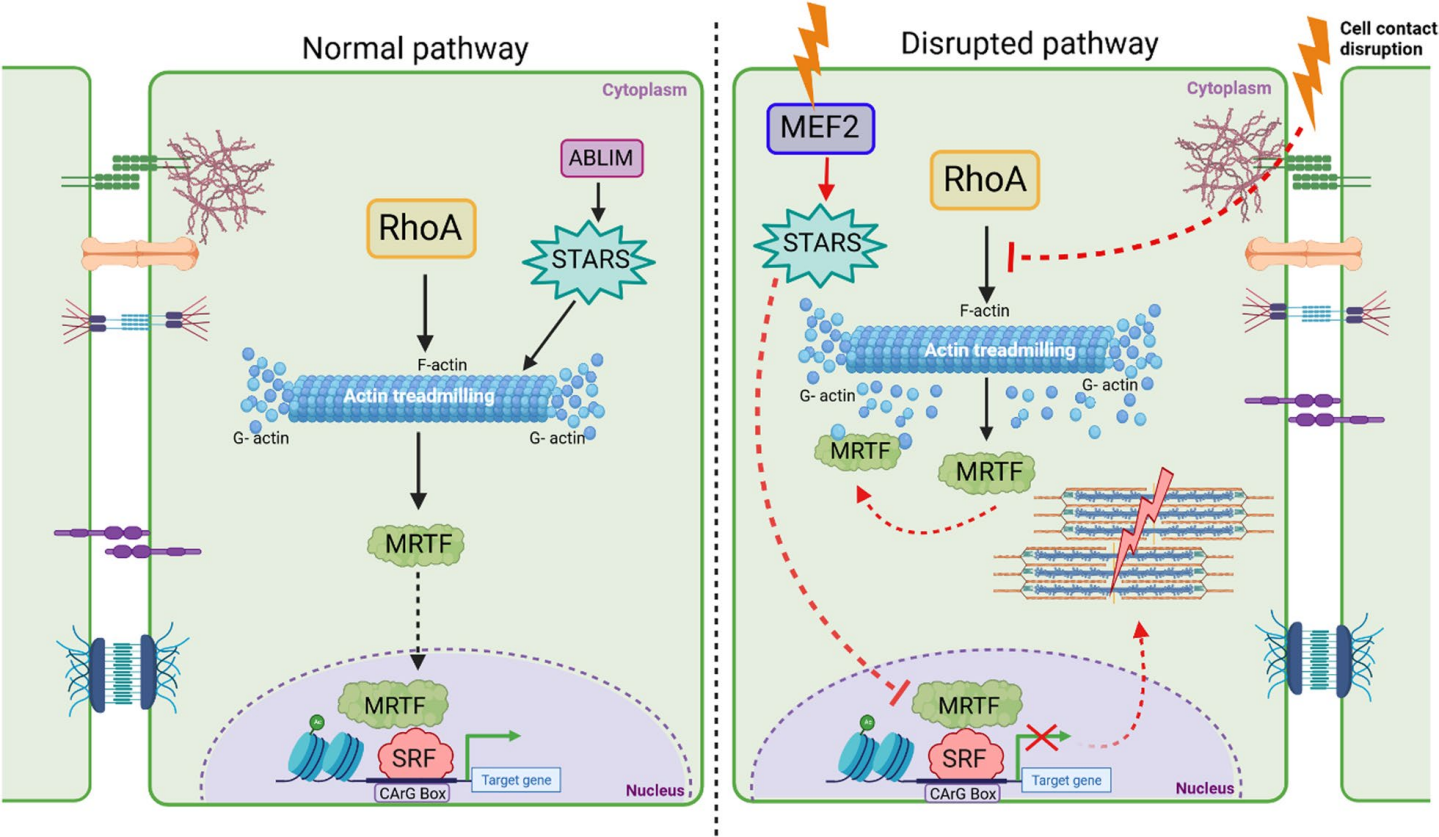
Impact of SRF transcriptional activation on the cytoarchitecture and cell–cell contact of cardiomyocytes- Under physiological conditions, SRF is activated upon RhoA-mediated activation, which is dependent on actin treadmilling. Striated muscle activators of Rho signaling (STARS) also facilitates this activation upon ABLIM stimulation. However, as seen in the right panel, when cell contact is disrupted, actin polymerization is inhibited, affecting actin treadmilling. In this case, G-actin-bound MRTF is retained in the cytoplasm, inhibiting SRF transcriptional activation and leading to structural destabilization because of sarcomere disruption. Furthermore, stress activates MEF2 expression, which inhibits MRTF-SRF activity via STARS. Thus, Rho plays a pivotal role in SRF activation. *RhoA* Ras homology family member A; *ABLIM* Actin binding LIM; *STARS* striated muscle activators of Rho signaling; *MRTF* myocardin related transcription factor; *SRF* Serum response factor; *MEF2* Myocyte enhancer factor 2A

The Rho family of GTPases consist of RhoA, Rac1 and cdc42 [81]. These Rho GTPases regulate multiple pathways to modulate the equilibrium of F-actin and G-actin levels in a cell. The state of F-actin is stabilized by Rho-associated protein kinase (ROCK). Upon activation of RhoA expression, monomeric G-actin is polymerized into F-actin filaments, MRTF-A is translocated from the cytoplasm to the nucleus, leading to further activation of SRF-mediated transcription (Fig. 5) [50, 82, 83].

Cell–cell contact is needed for RhoA-mediated control of the actin cytoskeleton, MRTF-A-SRF-mediated transcriptional activity and maintenance of the cardiomyocyte lineage [84]. E-cadherins linked to the actomyosin scaffold depend on actin dynamics mediated by the MRTF-SRF pathway [85, 86]. Migration of epicardial cells toward cardiac cells and SMCs depends on MRTF-A-SRF signaling [87]. MRTFs and SRF also exert special effects in endothelial cells, as indicated by selective disruption of MRTF-A, MRTF-B, or SRF resulting in defective angiogenesis [88, 89]. MRTF-B deletion leads to embryonic lethality due to reduced vascular SMC differentiation and malformations in the aortic arch, demonstrating the importance of the MRTF-SRF regulatory axis and its indispensability for epicardial cell mobility [87].

**Fig. 6:**
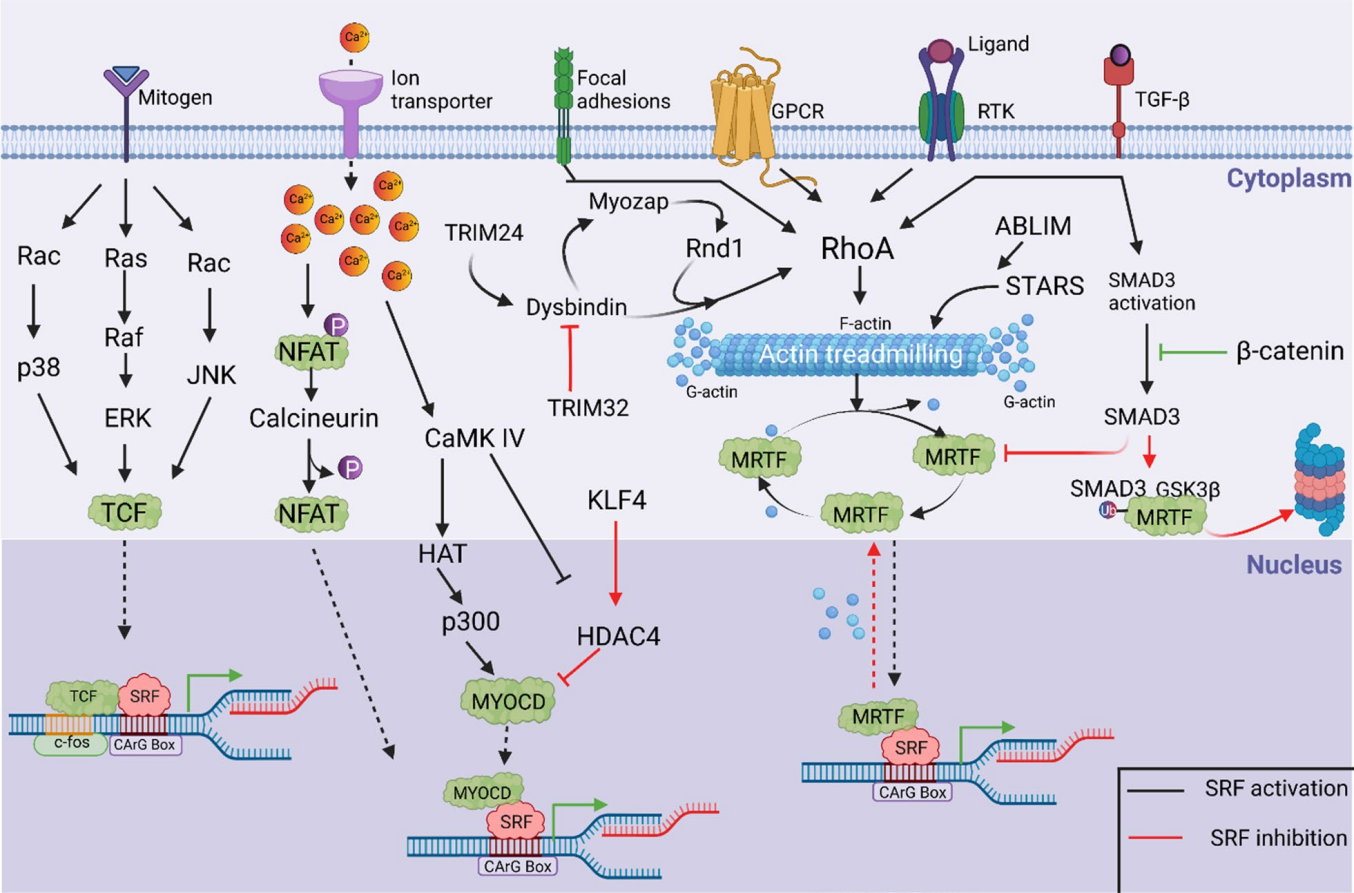
Molecular mechanisms of SRF signal transduction. Multiple signaling pathways exhibit an effect on SRF transcriptional activation. TCF-mediated activation is mediated by a mitogen stimulus through the Ras-Raf-MAPK pathway, which leads to the activation of SRF-mediated cell proliferation- and differentiation-related genes. In calcium signaling, NFAT is dephosphorylated by the phosphatase Calcineurin, which facilitates NFAT nuclear localization, suggesting firm binding of MyocD-SRF, which mediates transcriptional activation. Additionally, calcium signaling activates Ca^2+^/calmodulin protein kinase, which further activates histone acetyltransferases, thereby facilitating MYOCD-SRF activation; this interaction is inhibited by multiple other factors, such as KLF4, HOP, and HDAC4, with the nuclear localization of HDAC4 inhibiting SRF-mediated transcription. Homeobox protein (HOP) has been known to inhibit the myocardin-SRF interaction, inhibiting transcriptional inactivation. Reports have also shown that Elk1 (a member of the Ets family of transcription factors) competes with MYOCD to competitively bind to SRF, affecting SRF-induced transcription. In addition, multiple receptors, such as G protein-coupled receptors (GPCRs), focal adhesions, receptor tyrosine kinases, and TGF-β, influence SRF transcription via the RhoA-Actin-MRTF signaling cascade. Smad3 activation via TGF-β signaling inhibits MRTF nuclear translocation and ubiquitin-mediated degradation of MRTF via the Smad3 and GSK3β axes. Multiple other molecules, such as dysbindin, myozap, and Rnd1, have been known to affect SRF signaling via the Rho-A-MRTF-SRF axis. MRTF in its active form is transported from the cytoplasm to the nucleus to induce SRF activation. Monomeric G-actin binds and inactivates MRTF, which is retained in the cytoplasm in the inactive actin-bound state. Thus, actin treadmilling controls MRTF-mediated SRF transcriptional activity. Furthermore, STARS can activate MRTF-SRF by controlling G- to -F actin polymerization. Ras, rat sarcoma; Raf, rapidly accelerated fibrosarcoma; Rac, Ras-related C3 botulinum toxin substrate; cdc42, cell division control protein 42; JNK, c-Jun N-terminal kinase; TCF, ternary complex factor; SRF, serum response factor; Ca^2+^, calcium, NFAT, nuclear factor of activated T cells; HOP, homeobox proteins; CaMKIV, calcium calmodulin-dependent protein kinase 4; HAT, histone acetyl transferases; MyocD, myocardin; KLF4, Kruppel-like facto 4; HDAC4, histone deacetylase 4; GPCR, G protein-coupled receptor; RTK, receptor tyrosine kinase; TGF-β, transforming growth factor-beta; GEFs, guanine nucleotide-exchange factors; ROCK, Rho-associated protein kinases; RhoA, Ras homolog family member A; LIMK, LIM kinase, STARS: striated muscle activators of Rho signaling; MRTF, myocardin-related transcription factor; GSK3β, glycogen synthase kinase 3 beta; ABLIM, actin binding LIM; TRIM24, tripartite motif containing 24; TRIM32, tripartite motif-containing 32; and P, phosphorylation

*Cell–cell contact and cell fate* Maintenance of cell–cell contact at intercalated discs of a cardiomyocyte is one of the functions of RhoA [84]. Compromised cell–cell contact at intercalated discs impedes Rho-mediated actin polymerization [84], leading to increased cytosolic free G-actin, which binds to MRTF and prevents it from undergoing nuclear translocation [84]. Thus, entire MRTF-SRF-mediated myogenic gene activation process is terminated, and this termination switches the cardiomyocyte fate to express the adipocyte gene program [84]. Notably, myocardium-enriched **Z**o-1-inter**a**cting **p**rotein (Myozap) is an intercalated disc protein known to interact with and induce RhoA-SRF signaling [90, 91]. Myozap-overexpressing mice developed cardiac hypertrophy, whereas mice lacking Myozap expression showed a hypertrophic response but only when subjected to mechanical stress [90, 92]. Modulation of MAPK-SRF signaling is a key factor in stress-induced hypertrophy [92, 93]. In addition, some interactors of Myozap, namely, dysbindin and Rnd1, regulate the RhoA-SRF axis in cardiomyocytes [92–94]. Initially studied in the schizophrenia, dysbindin upregulates the expression of SRF target genes Acta1 and Actc1 and, upon overexpression, induces a hypertrophic response (Fig. 6) [90, 93, 95]. Furthermore, we recently identified SH3-binding glutamic acid rich (SH3BGR) to be an inducer of RhoA-SRF signaling in NRVCMs [96].

#### Transforming growth factor-beta (TGF-β)

Certain factors exert effects on the MRTF-SRF axis, including transforming growth factor-beta (TGF-β) through signaling and integrins via Rho and Rac GTPase activity [85]. β1 integrin regulates Rho-mediated activation of α-actin in cardiomyocytes [97]^.^ TGF-β, is a family of cytokines consisting of TGF-β polypeptides, activin and BMP, modulates Rho GTPase-mediated actin dynamics and activates MRTF-SRF signaling [85, 98–100]. One of the targets of TGF-β activation, Smad3, binds to MRTF-A and induces its ubiquitin-mediated degradation by recruiting GSK3β [101]. Disruption of intercellular junctions or activation of TGF-β activates β-catenin, leading to Smad3 sequestration and MRTF-A release, which induces SRF-mediated myogenic gene expression (Fig. 6) [101, 102].

*Cell fate* The importance of cell–cell contact in the maintenance of cardiomyocyte fate has been established. Adipocytes and myocytes share a common mesenchymal progenitor lineage. SRF negatively regulates brown adipogenesis by inhibiting TGF-β-BMP signaling pathway activation [103]. Therefore, it has been hypothesized that equitable activation of SRF is the key to maintaining the distinction between a cardiomyocyte and adipocyte fate. Interestingly, epicardial fat, which protects the heart under physiological conditions, is derived from brown adipose tissue [104]; nevertheless, epicardial fat is associated with obesity-related coronary artery disease [105]. These cross-talks are still not fully elucidated, which opens up a variety of opportunities to study molecular signaling in greater detail and provide clues to the ubiquitous role played by SRF in the heart and vasculature.

#### Adenylyl cyclase-associated protein 2 (CAP2)

CAPs are actin monomer-binding proteins critical for the depolymerization of actin in cells [106]. Loss of CAP2, one of two known CAP proteins, with the other being CAP1, affects cellular actin treadmilling [106]. The expression levels of the SRF target proteins Acta2 and Myl9 were elevated in CAP2-knockout cardiac cells in mice [106]. Inhibition of SRF expression in these mice reversed this effect and resulted in prolonged survival, suggesting that cytoskeletal stress-mediated SRF upregulation may be detrimental to the heart. These studies suggest an important function of the MRTF-SRF interaction in cardiac cellular homeostasis.

#### Striated muscle activators of Rho signaling (STARS)

STARS are muscle-specific proteins that bind to actin, stimulating SRF activity via the nuclear localization of MRTF-A and MRTF-B after F-actin stabilization [107, 108]. Although some reports have emphasized the importance of STARS expression for normal cellular function, conflicting reports have indicated that STARS is involved in adverse cardiac remodeling [116]. Localized to Z-discs, STARS modulates cardiac responsiveness to stress signals by acting as a cytoskeletal mediator of the interaction between Myocyte enhancer factor 2 (MEF2), a stress responsive transcription factor, and SRF [116]. MEF2 has a direct target, myotonic dystrophy protein kinase (DMPK), which causes sarcomere degradation by phosphorylation-based inactivation of SRF [109]. STARS activity is enhanced by two actin-binding (AB) LIM family proteins, namely, ABLIM2 and ABLIM3 [117]. GATA4 is also a regulator of STARS activity [118]. Interestingly, disruption to STARS activation resulted in cardiomyocyte hypertrophy [118]. An in silico study was performed via comparative genomics to determine the effect of a gain- or loss-of-function of STARS [118]. The results indicated that GATA4 repressed STARS activity in embryonic, neonatal, and adult hearts [118], and this inhibition may exert a major effect on the MRTF-SRF axis. However, further in vitro and in vivo studies are needed to substantiate these claims. STARS expression knockdown in zebrafish resulted in severe contractile dysfunction which was reestablished by SRF [110]. STARS plays a crucial role in the development of acute pulmonary hypertension by increasing the proliferation of pulmonary atrial SMCs through the activation of the SRF/Egr1 pathway [119]. Overall, these studies demonstrated that STARS is both beneficial and detrimental to the cardiovascular system, although the exact circumstances and mechanisms supporting these findings remain unclear.

#### Yin Yang 1 (YY1)

YY1, a zinc family protein and member of the GLI-Kruppel family, binds to CArG boxes and functions as a transcriptional activator, repressor or initiator [120–122]. YY1 is a negative regulator of SRF activity [120]. Specifically, YY1 and SRF compete for binding to the CArG box of cardiac α-actin and many other muscle-specific gene promoters, and apparently differences in the sequence that binds the CArG boxes differs only slightly in YY1 and SRF [113]. Another mode of suppression involves SRF interacting with myocardin, disrupting the myocardin-SRF complex [123]. Enhanced expression of YY1 in damaged carotid arteries suggested that YY1 plays a role in inhibiting vascular SMC differentiation, with trauma characterized by a sharp decrease in vascular SMC growth and differentiation [123].

#### Four and half LIM proteins (FHL), Titin and TRIMs

Four and half LIM proteins (FHL-1 and FHL-2) are multifunctional proteins abundant in cardiomyocytes and localized in association with titin to sarcomeres [124]. FHL2 negatively affects the MAPK pathway by suppressing its activation mediated through ERK, thereby preventing its nuclear translocation and activation [125]. Similarly, FHL2 has been observed to exert antagonistic effects on the Rho/MRTF-A-mediated SRF signaling pathway [112]. FHL2 is not only a SRF target gene but also an interactor of SRF both in vitro and in vivo [112, 125]. Mice with FHL2 -knockout cardiac cells exhibited normal functioning, but upon stimulation with prohypertrophic agents, mouse cardiac abnormalities were evident [112]. FHL2 was translocated to the nucleus in a Rho-dependent manner and competed with MRTF-A for SRF binding [112]. However, in contrast to MRTF, FHL2 inhibited SRF activity, suggesting an inhibitory feedback effect of FHL2 on SRF in cardiomyocytes [112]. In Xenopus embryos, FHL2 suppressed VEGF-induced angiogenesis [111].

In addition, titin, a major sarcomeric protein, plays an essential role in cardiomyocytes and is crucial for the flexibility- and stretch-related mechanisms in myocytes [114]. Titin contains a protein kinase domain (TK) that senses the mechanical loading of a cell. The TK domain interacts with the zinc finger protein Nbr1 (an autophagy-related protein in muscle cells). Nbr1 directs ubiquitin-associated p62/SQSTM1 to a sarcomere, and p62 interacts with MuRF2/TRIM55 [114]. TRIM55 is a muscle-specific ring B box E3 ubiquitin ligase and acts as a ligand with affinity for the transactivation domain in SRF [114]. The nuclear translocation of TRIM55, induced by low mechanical load, reduces SRF presence in the nucleus, thereby repressing SRF-mediated transcription [114]. Human mutations in the TK domain of Titin affect the Titin-p62-TRIM55-SRF axis and cause hereditary muscle diseases with dissociated sarcomeric structures [126]. Similarly, other TRIMs, such as TRIM24 and TRIM32, have been linked to SRF signaling. As mentioned earlier in this review, TRIM24 together with Dysbindin activates SRF via RhoA; on the other hand, TRIM32 causes Dysbindin degradation, thereby inhibiting SRF signaling [115]. Although multiple TRIM family proteins have been shown to be involved in various cardiac diseases, the roles they play and the extent of their association with SRF remain to be explored [127].

#### TEA domain transcription factor 1 (TEAD) and yes-associated protein 1 (YAP)

TEAD and YAP are known transcription factors and cofactors, respectively, in the Hippo pathway. These molecules contribute to cardiac fibrosis via myocardin and/or MRTF. Nkx2.5 binds to the myocardin promoter, and the resulting increase in Myocardin expression supports the differentiation of vascular SMCs derived from cardiovascular progenitor cells [128]. However, YAP sequesters Nkx2.5, inhibiting myocardin-mediated differentiation [129]. YAP1 switches the contractile phenotype of a cell to one in which SMCs proliferate because of downregulated SRF-myocardin axis signaling [130]. YAP expression in fibroblasts increases upon myocardial injury and causes myofibroblast differentiation together with increased TEAD1 levels by upregulating MRTF-A expression. Both genetic and pharmacological ablation of YAP leads to fibroblast remodeling, attenuating cardiac fibrosis [131]. By affecting SMC gene expression, TEAD1 plays a temporal role. During embryonic stages, TEAD1 induces vascular SMC and cardiac gene expression by promoting myocardin and paired-like homeodomain 2 (Pitx2) expression; however, in adults, TEAD1 suppresses SMC gene expression by disrupting the myocardin- SRF complex [132, 133]. Thus, inhibition of YAP may be a potential target to study, but the direct interaction of the Hippo and SRF signaling pathways remains elusive and requires further study.

#### MicroRNAs (miRNAs) and long noncoding RNAs (lncRNAs)

Recent studies have elucidated the roles played by miRNAs in muscle gene expression, actin dynamics and stress responses, as well as the MRTF-SRF axis [4]. SRF activates two bicistronic miRNAs, namely, miR1-1 and miR133a, which regulate many mRNAs of MRTF-SRF target genes [134]. miR133 suppresses SRF expression, inhibiting the expression of certain muscle genes through feedback inhibition [135]. In addition, miR143 and miR145, which are expressed in the embryonic heart derived solely from the SMC lineage, are essential activators of myocardin [136, 137]. The absence of miR143 and miR145 leads to the expression of SRF targets after injury, and miR143- and miR145-null mice are resistant to vascular remodeling due to defective vessel wall formation and malfunctioning cytoskeletal proteins [136]. miR143 and miR145 miRNAs facilitate important cytoskeletal activities, such as activation of cofilin, promotion of cell mobility by polymerizing F-actin, and modulation of small GTPase activity for proper cellular function [136]. Disruption to the expression of these miRNAs contributed to the disruption of the MRTF-SRF axis, impacting the cytoskeletal structure and impairing SMC homeostasis [136]. Another miRNA important to cardiac and skeletal muscle cell functions is miR486 [138]. As a downstream effector of MRTF-SRF, miR486 promotes PI3K-AKT signaling and inhibits phosphatase and tensin homolog (PTEN) and forkhead box protein O1A (FOXO1A) expression, which are negative regulators of PI3K signaling [138]. An increase in miR486 concentration in response to mechanical stress promotes cardiomyocyte proliferation and growth by increasing SRF levels via a feedback loop [139]. These findings indicate a new modality modulating the MRTF-SRF axis by synchronizing miRNA expression to sustain cellular homeostasis [138].

In addition to miRNAs, lncRNAs play roles in SRF activity regulation. Noncoding RNAs comprise a newly discovered class of transcriptional regulators that act in a tissue-specific manner. Recent studies have indicated that lncRNAs regulate SRF-mediated gene activation in cardiomyocytes. For example, a lncRNA (CARDINAL) located upstream of myocardin has been reported to robustly activate the cardiac transcription factors MEF2 and Myocd/MRTF [140]. Localized to chromatin in cardiomyocytes, CARDINAL is critical for inhibiting TCF-mediated SRF activation by forming a complex with SRF. Knocking out CARDINAL expression in mice led to increased SRF-mediated mitogenic gene expression and decreased heart function with increasing age and stress and after ischemic injury. Furthermore, CARDINAL and Myocd expression was significantly upregulated during heart failure in mice and humans, suggesting that they regulate the SRF-mediated cardiac gene network critical to proper functioning of the heart and ventricular remodeling due to stress [140]. Another lncRNA named MYOcardin-induced smooth muscle long noncoding RNA, inducer of differentiation (MYOSLID), a transcriptional target of Myocd/SRF, hampered vascular SMC cell proliferation and induced SMC differentiation [10]. MYOSLID is a direct transcriptional target of Myocd/SRF and TGFβ/SMAD, and loss of MYOSLID abrogated the nuclear translocation of Myocd-related transcription factors and TGFβ signaling, affecting SMC proliferation and differentiation [141]. Considering the aforementioned pathways involved in SRF signaling and regulation, we conceptualize SRF and its transcriptional activation as a complex process. In the journey from the EMT to the formation of cardiac lineages and the maintenance of cardiomyocyte homeostasis in adults, SRF plays an important role, and these processes are disrupted when SRF is dysfunctional or missing. Thus, SRF can be considered a critical factor in cardiac development and homeostasis.

## Significance of SRF in cardiovascular diseases

The aforementioned sections illustrated the involvement of SRF and its cofactors in processes essential for cardiac development and homeostasis. Disruption at any level of the SRF axis results in undesirable outcomes. However, what roles does SRF play in cardiovascular diseases? Do all cardiac diseases exhibited disrupted SRF or SRF-related molecules? Or is SRF disruption a disease-specific sign? A study comparing nonfailing and failing hearts suggested that failing hearts exhibited a 40% increase in the expression of SRF Δ4,5 (~ 52 kDa) accompanied by a drastic decrease in full-length SRF [10, 142]. Hence, the authors stated that the truncated isoform of SRF was the key factor in inducing heart failure [10, 142]. Additionally, one of the characteristics of a failing heart was found to be an elevated level of caspase 3 [143]. SRF is cleaved by caspase 3, and the cleaved 32-kDa N-terminal SRF protein is a cause of heart failure [143]. Similarly, SRF cleavage by enteroviral protease 2A has also been predicted to play a role in cardiomyopathy [144]. Enteroviral protease 2A cleaves SRF at the transactivation domain, creating an ~ 50-kDa N-terminal SRF molecule with impaired DNA-binding capacity [144]. To determine whether the truncated version or the cleaved version is critical for heart failure, further study is required. Other cardiovascular diseases, such as age-related hypertension and corresponding arterial stiffness, also involve SRF activity. The addition of Y-27632, a selective inhibitor of ROCK expression, or CCG‐100602, a SRF/myocardin inhibitor, reduced overall arterial stiffness as the pathological condition was Rho/ROCK/myocardin/SRF-mediated [145–147]. Since ROCK operates upstream of the myocardin-SRF axis, the effects of Y-27632 were broad and included various undesirable effects, whereas CCG‐100602-dependent myocardin/SRF inhibition was specific, attenuating arterial stiffness [145, 146] [147].

Diseases are characterized not only by transcript variants but by mutations that also play a part under some conditions. One of the latest examples involves one of the two mutations 821A > G and 880G > T in the SRF gene coding sequence in sporadic conotruncal heart defect patients [148]. Strikingly, both the mRNA and protein levels of SRF were unaffected by these mutations; however, the resultant mutant proteins lacked SRF transcriptional activity [148]. The loss of SRF transcriptional activity may be one of the causes for the heart defect in these patients [148].

The phosphorylated state of SRF plays a crucial regulatory role in cardiac diseases [149]. For instance, phosphorylation of SRF due to the muscle A-kinase anchoring protein β (mAKAPβ) and ribosomal protein S6 kinase A2 (RSK3) interaction leads to pressure overload-induced concentric hypertrophy, while dephosphorylation of SRF due to the mAKAPβ and protein phosphatase 2A (PP2A) interaction leads to dilated cardiac hypertrophy [149]. Certain adeno-associated virus (AAV) can mediate the delivery of anchoring-disrupting peptides to obstruct individual complex formations that can help attenuate respective pathological conditions [149]. These findings suggest new dimensions toward understanding the importance of the SRF interactome in cardiac functional equilibrium. The diseases in humans caused by SRF modulation are summarized in Table 4.

**Table 4:** Summary of disease conditions caused due to SRF modulations

Disease	Causes	References
Heart failure	Increased levels of truncated SRF isoform	[10, 142]
	Cleavage of SRF by Caspase 3	[143]
Dilated cardiomyopathy	Cleavage of SRF by enteroviral protease 2A	[144]
	Decreased SRF Ser^103^ phosphorylation	[149]
Hypertension and arterial stiffness	Vascular smooth muscle cell stiffening due to induction of SRF signaling	[145–147]
Conotruncal heart defect	Loss of transcriptional activity due to SRF gene mutations, 821A > G or 880G > T	[148]

The role of SRF in cardiac diseases is not always detrimental. Connective tissue growth factors (CTGFs) are profibrotic cytokines crucial for cardiac fibrosis-mediated dilated cardiomyopathy (DCM) and heart failure by promoting fibroblast proliferation [150]. SRF-deficient mice presented with increased expression of CTGF and hearts that mimicked DCM [150]. Cardiac-specific insulin growth factor (IGF) supplementation downregulated the expression level of CTGF in cardiomyocytes, thus inhibiting CTGF secretion [150]. Abrogated CTGF secretion blocked paracrine signaling-mediated fibroblast proliferation [150]. IGF counteracted the inflammatory response and fibrosis in mutant mice, protecting the heart against adverse cardiac remodeling and DCM [150]. Thus, supplementation with cardiac IGF1 may reverse DCM caused by SRF depletion to some extent; however, further insights are needed before IGF1 can be used in therapeutics [151].

SRF cofactor modulation can also result in the disease phenotype acquisition. For instance, MRTF-A is a cofactor that controls the expression of extracellular matrix proteins such as collagen and elastin postmyocardial injury. MRTF-A-null mice presented with a reduced number of myofibroblasts, indicating sustained injury insult [152]. Can inhibiting MRTF-A alone be a post-myocardial injury treatment for cardiac fibrosis? In addition, myocardin levels are drastically decreased after ferric citrate treatment[153], and ferric citrate is administered to patients with chronic kidney disease (CKD) postdialysis to regulate blood iron levels. When administered 4% ferric citrate, mice with CKD exhibited decreased blood pressure, diminished inflammation and fibrosis and reduced CKD-induced hypertrophy [153]. Is myocardin the main factor in CKD-induced cardiac hypertrophy and fibrosis? If it is, how does ferric citrate downregulates myocardin?

SRF or its cofactors may attenuate deleterious effects of the cardiac pathophysiology. From mutant isoforms of SRF to overexpression or deletion of its interaction partners, SRF has been shown to play a role in a plethora of cardiovascular diseases, such as cardiac fibrosis, cardiac hypertrophy, hypertension and ultimately heart failure. This highlights the important role a single transcription factor plays, governing effects at various levels, in a cell.

## Conclusions and future perspectives

Overall, SRF is a very important transcription factor involved in a multitude of cellular functions. The involvement of many other molecules in SRF function reveals the complexity of this interesting factor. The combined effect of modulators and cofactors with SRF in cytoskeletal remodeling determines cell fate [84, 103]. Similarly, cell contractility [39, 110, 130], intracellular contacts [18, 84, 85, 90], actin dynamics and sarcomere organization are strictly governed by SRF and its cofactors. SRF regulates cell metabolism by maintaining mitochondrial dynamics, fatty acid translocation and the expression of electron transport chain (ETC) complex proteins. Epigenetic modifications are major components affecting the activation of genes downstream of SRF. SRF is significance in developmental stages, and an adequate concentration of SRF is essential for cardiovascular homeostasis.

SRF activity is involved in the pathological conditions of organs in addition to the heart. For example, cerebral artery-derived vascular SMCs from Alzheimer's patients exhibited enhanced levels of myocardin/SRF [154]. These elevated levels were correlated with the hypercontractility of small cerebral arteries caused by elevated concentrations of contractile proteins in vascular SMCs [154]. As expected, ex vivo aortic rings exhibited increased contractility with myocardin overexpression [154]. This outcome was correlated with coronary spasms characterized by increased vessel contraction that provoke heart attacks. In addition, SRF plays a role in the homeostasis of the gastrointestinal (GI) tract [155]. Severe developmental defects were observed in the GI tract of SRF-deficient mice [155]. The absence of SRF in SMCs of both human rectal prolapse tissue and partially obstructed intestinal tissue suggested that SRF plays a role in GI tract pathology [155]. With respect to renal cells, SRF stimulates EMT-mediated dysfunction in tubular epithelial cells in both diabetic and hyperuricemic nephropathy [156, 157]. SRF is also an early marker of acute kidney injury [158].

The expression of many SRF cofactors is upregulated under pathological conditions. It is possible that the SRF axis is the default pathway that is activated upon myocardial stress. Modulating a few upstream genes/proteins of certain upregulated SRF cofactors upon cardiac dysfunction may be beneficial. For every single function of SRF, there might be a regulatory mechanism in which a cofactor is needed at a specific time. Similarly, a specific type of cell stress might activate a specific SRF-cofactor axis, and understanding these complex mechanisms might lead to a cure for a specific disorder.

The multifactorial nature of diseases and the multidimensional functions of individual molecules or pathways are reasons that targeting these molecules for preventive/treatment can be either useless or harmful. However, prevention or treatment of a pathological conditions can still be achieved in multiple ways. First, SRF or its cofactors can be targeted by either pharmacological inhibitors [145–147], disruption of the SRF DNA-binding domain, or a decoy oligonucleotides devised for reducing abnormal gene expression during pathological conditions [159]. For instance, the MHD domain in cardiac myocardin exerts an autoinhibitory effect, which is enhanced by a rare lysine-to-arginine mutation that attenuates cardiomyocyte hypertrophy [160]. Exploring and making use of these characteristics may lead to the development of therapeutic interventions. In addition, molecules can be used to disrupt SRF-associated interactions; for instance, peptides that obstruct complex formation can be delivered into cells [149]. Furthermore, a disease phenotype can be targeted; for example, supplementation with IGF1 can reverse the DCM phenotype [150]. In addition, *out of the box* approaches can be applied. Previously described as *transcriptional noise*, lncRNAs have been used in various fields. Notably, a p53-binding lncRNA was used to prevent p53 activation of myocardin-mediated autophagy, preventing cardiomyocytes from undergoing autophagy-related apoptosis and ischemic/reperfusion injury [161].

Preventives and cures are critical, and the development of either a preventative or cure depends on thorough investigation and exploration. Despite the cell type-dependency of many molecular functions, acknowledging and integrating differences leads to novel perspectives. For example, in cardio-oncology, an emerging field focused on the identification, prevention and treatment of cardiovascular issues related to cancers and cancer therapies, such as targeted chemotherapy, radiation therapy, and immunotherapy [162]. Cancer cachexia, a muscle-wasting disorder, for example, results in cardiac function impairment [163]. However, we have yet to understand how cancer-driven cardiac defects alter SRF levels/activity. Knowing the indispensable role played by SRF in muscle and cardiac physiology, we believe that SRF is a promising molecule to exploit for its therapeutic potential in cancer or cardiac cachexia. When the findings of diverse studies are combined, multiple aspects of the findings are events, and connecting the dots is pivotal. Specifically, finding the missing link between TGF-β signaling and SRF with respect to the change in the fate of cardiomyocytes into adipocytes is an interesting topic of study [105]. Notably, this line of inquiry adds the prospect of a role played by SRF not only in the cardiovascular system but also in association with epicardial fat. Hypothetically, SRF may be critical, at least in part, for the comorbidities associated with obesity and cardiovascular diseases; however, more substantiating evidence needs to be collected.

Although research over nearly three decades has revealed many facets of SRF, we believe that many unexplored aspects still require attention. The bases of heart disease are the molecular modulators and signaling cascades at the cellular level that lead to the regulatory activities that impact a whole living system. It is not surprising that these molecules are interwoven into the complex human system; this complexity helps us appreciate the level of effort needed to help humanity reach new heights by devising and continuously improving therapeutic approaches to treat diseases.

Despite many questions, there are very few answers, and the only way to close the knowledge gap is performing more research.

## Data Availability

Not applicable.
